# Mitochondrial Genome Editing to Treat Human Osteoarthritis—A Narrative Review

**DOI:** 10.3390/ijms23031467

**Published:** 2022-01-27

**Authors:** Gang Zhong, Henning Madry, Magali Cucchiarini

**Affiliations:** Center of Experimental Orthopaedics, Saarland University and Saarland University Medical Center, Kirrbergerstr. Bldg 37, D-66421 Homburg, Saar, Germany; 13257788667@163.com (G.Z.); henning.madry@uks.eu (H.M.)

**Keywords:** human osteoarthritis, mitochondria, mitochondrial DNA, genome editing

## Abstract

Osteoarthritis (OA) is a severe, common chronic orthopaedic disorder characterised by a degradation of the articular cartilage with an incidence that increases over years. Despite the availability of various clinical options, none can stop the irreversible progression of the disease to definitely cure OA. Various mutations have been evidenced in the mitochondrial DNA (mtDNA) of cartilage cells (chondrocytes) in OA, leading to a dysfunction of the mitochondrial oxidative phosphorylation processes that significantly contributes to OA cartilage degeneration. The mitochondrial genome, therefore, represents a central, attractive target for therapy in OA, especially using genome editing procedures. In this narrative review article, we present and discuss the current advances and breakthroughs in mitochondrial genome editing as a potential, novel treatment to overcome mtDNA-related disorders such as OA. While still in its infancy and despite a number of challenges that need to be addressed (barriers to effective and site-specific mtDNA editing and repair), such a strategy has strong value to treat human OA in the future, especially using the groundbreaking clustered regularly interspaced short palindromic repeats (CRIPSR)/CRISPR-associated 9 (CRISPR/Cas9) technology and mitochondrial transplantation approaches.

## 1. Introduction

Osteoarthritis (OA) is the most prevalent chronic joint disorder that affects millions of adult individuals worldwide [1,2]. The progressive, irreversible degradation of the extracellular cartilage matrix (ECM) with the persistence of chronic inflammation are all hallmarks of OA [3,4,5,6,7]. Current treatments against OA mostly target pain and inflammation or address cartilage degeneration via surgical interventions for severe and end-stage cases of the disease, but none of them is capable of fully reproducing a natural cartilage surface and joint integrity [8,9], showing the critical need for improved therapeutic approaches to cure OA.

OA has also been described as a mitochondrial disorder in light of findings showing a deterioration of the functions of these central cell organelles in OA chondrocytes during the development and progression of the disease [10]. Such mitochondrial dysfunction is associated with the presence of mutations in the peculiar DNA of the mitochondria (mtDNA) [10,11] that codes for proteases supporting mitochondrial oxidative phosphorylation processes [12], leading to increased oxidative stress and contributing to alterations in the chondrocyte phenotype and, ultimately, to cartilage degeneration [13,14].

Exploring novel ways to address the pathology of OA based on the perception that OA is also a mitochondrial disease may help define improved treatments to cure this severe disorder. The emergence of genome editing technologies that may be applied to target the mitochondrial genome provides hope to precisely modulate mtDNA genes involved in the development of OA as a means to reliably manage this disease in patients. Such innovative mitochondrial treatment options for OA are being discussed in this narrative article with the goal of feasible and safe translational applications in the near future.

## 2. Osteoarthritis

Osteoarthritis (OA) is the most common chronic disease of large joints, such as the knee, hip and hand joints, that mostly occurs in elderly individuals, with an incidence of greater than 60% in individuals over 65 years of age and that may further rise within the next 30 years [1,2]. OA does not only shatter joint functions and physical abilities but also brings long-term, considerable pain in patients [8]. The risk factors for OA include primary (gender, genetic inheritance, obesity, hormonal changes) and secondary (trauma, mechanical stress, metabolic changes) components [7]. The features of OA include the degeneration of the articular cartilage, synovial inflammation, subchondral bone sclerosis and osteophyte formation [3,4,5,6,7].

The healthy articular cartilage consists of a layer of hyaline cartilage devoid of vascularisation, the lymphatic system and innervation, covering the end of bones in the joint to resist shocks and withstand mechanical loading and impact [15]. Under pathological conditions in OA, the chondrocytes undergo phenotypic changes and alterations in their homeostasis in response to an accumulation of detrimental pro-inflammatory mediators (interleukin-1 beta—IL-1β, tumour necrosis factor alpha—TNF-α, nitric oxide—NO, prostaglandin E2—PGE2, reactive oxygen species—ROS, etc.) and matrix-degrading enzymes (matrix metalloproteinases—MMPs, A disintegrin-like and metalloproteinase with thrombospondin motifs—ADAMTS, etc.), leading to the loss of ECM elements (proteoglycans, type-II collagen) [3,4,5,6,7].

The current drugs against OA mainly focus on analgesia and inflammation (celecoxib, rofecoxib), providing temporary relief symptoms, but they are not capable of preventing the progression of the disease [8,9] and may exert serious side effects in the gastrointestinal and blood systems [16]. Other treatments include the use of joint lubricants (hyaluronic acid, glucosamine sulphate, chondroitin sulphate) to reduce frictions on damaged cartilage surfaces [8], but their efficacy remains controversial [17]. For severe and end-stage cases of OA, surgical procedures may be attempted (fusion, debridement, osteotomy, joint replacement), but they lead to a considerable loss of quality of life, aside from the economic burden [9].

These findings show the crucial need to identify novel pathomechanisms of the disease that may lead to the generation of improved, more effective therapeutic options for OA.

## 3. Implication of the Mitochondria in Osteoarthritis

Accumulated evidence indicates that the nutrient supply of the articular cartilage is provided by the synovial fluid [18]. Independently from the oxygen blood supply, the chondrocytes have been traditionally perceived as highly glycolytic cells, obtaining energy from anaerobic respiration of glucose, a notion explaining that, until recently, the mitochondria were long neglected in the pathophysiology of OA [19,20]. However, up to 25% of articular cartilage energy needs to be met by high-energy electron transfer across the inner mitochondrial membrane during oxidative phosphorylation, a percentage pulled up under the high energy demand of the tissue [21,22]. In fact, nutrients and oxygen in the synovial fluid supply chondrocytes mainly through differential penetration in the ECM, depending on their molecular size, charge and depth of penetration [10]. As a result, this minimal oxygen partial pressure is maintained in the deep layer of the cartilage, where the glycolysis of chondrocytes and even of mesenchymal stem cells (MSCs) contributes much more to energy metabolism than to aerobic respiration. Chondrocytes and MSCs maintain an oxidative phosphorylation-independent survival after long-term evolutionary adaptation, and MSCs exhibit an environment-adaptive chondrogenic differentiation potential [23]. The surface layer of the cartilage can receive 5–7% oxygen, which is enough for the chondrocytes to carry out sufficient oxidative phosphorylation undertaken by the mitochondria [18].

Mitochondria are organelles with a double-layer membrane, converting various forms of energy into intracellular energy in the form of adenosine triphosphate (ATP) via ATPases and with the participation of high-energy electrons in the electron transport chain of the inner mitochondrial membrane through the process of oxidative phosphorylation [24,25]. ATPase binding proteins are responsible for material exchange and signal transmission with other cellular compartments, especially the nucleus, branching out the metabolism and biological behaviour of the mitochondria [26].

Mitochondria have an independent, small circular double-stranded DNA (mtDNA) encoding 13 proteins, 22 transfer RNAs (tRNAs) and 2 ribosomal RNAs (rRNAs) [27] that contribute to oxidative phosphorylation processes [28]. The complexity of the mitochondrial genome is reflected by the multiple copies of mtDNA where wild-type and variant mtDNAs coexist in heteroplasmy [29,30].

mtDNA is susceptible to exogenous stimuli (ultraviolet radiation, radioactive substances) and endogenous factors (by-products of aerobic respiration, ROS, RNS) that may lead to high-rate mutations. Mitochondria have nuclear-like DNA repair mechanisms to withstand mtDNA damage, including base excision repair (BER), direct reversal (DR), mismatch repair (MMR), cross-damage repair synthesis (TLS) and double bond break repair (DSBR) [31,32]. Nevertheless, the repair effects are fragile and balancing mitochondrial homeostasis in extreme environments remains challenging [31]. Accumulated DNA damage will, thus, cause lasting mitochondrial dysfunction as well as a pathological shift in the mitochondrial phenotype when the accumulation of damaged mitochondria reaches a threshold [33].

During the pathogenesis of OA, the mitochondria display important abnormal features in diseased chondrocytes compared with healthy cells, showing an increase in mitochondrial mass, decreased activities of the respiratory complexes II and III, reduced capacities of antioxidant enzymes (superoxide dismutase—SOD, glutathione peroxidase—GSH) and incomplete mitochondrial electron transfer resulting in excessive ROS and RNS production consuming high-energy electrons and greatly reducing the production of ATP [10,34,35]. The accumulation of ROS and RNS subsequently damages the mtDNA and mitochondrial membrane proteins, challenging the metabolism of the nucleus and of the entire chondrocytes [13].

**Figure 1:**
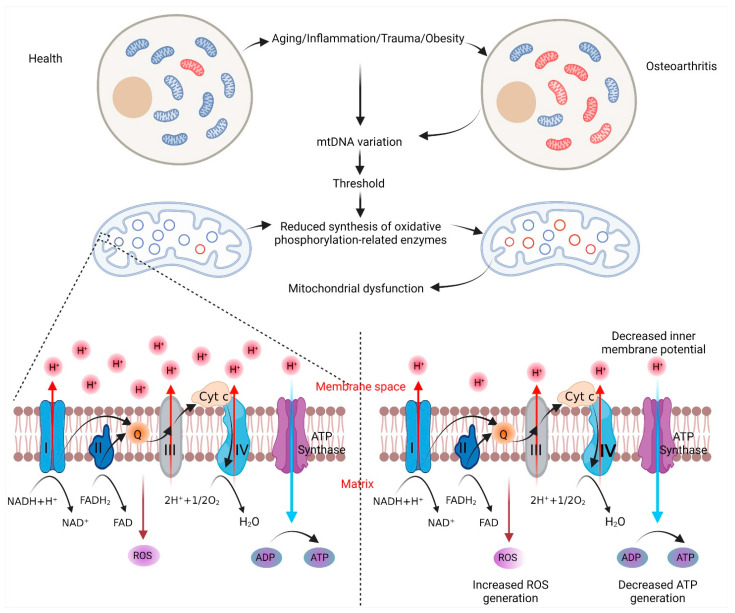
Influence of mtDNA heterogeneity and mitochondrial functions in OA. Under the influence of various risk factors, mtDNA mutations are observed in OA, affecting the synthesis of oxidative phosphorylation-related enzymes. Reduced synthesis of these enzymes leads to a poor transfer of high-energy electrons, which, in turn, reduces the production of ATP, increases the production of ROS and reduces the mitochondrial membrane potential. Increased ROS can subsequently aggravate the oxidative damage of chondrocytes, leading to cartilage degradation. Abbreviations: OA, osteoarthritis; mtDNA, mitochondrial DNA; ATP, adenosine triphosphate; ROS, reactive oxygen species; NAD, nicotinamide adenine dinucleotide; FAD, flavin adenine dinucleotide; Cyt c, cytochrome c; ADP, adenosine diphosphate (created with BioRender).

Mutations in the mtDNA have been reported during the pathology of the disease [10,11] (Figure 1), with a minimum prevalence rate of 20 per 100,000 [36], generally accompanied by reduced mtDNA repair capacity, decreased cell viability and increased apoptosis in OA chondrocytes [37,38]. Moreover, conducting systematic genetic screening of OA cases, Chang et al. [39] evidenced age-dependent gene deletions in the 4.977-bp mtDNA related to the process of idiopathic knee joint OA.

In light of such findings, the manipulation of the mtDNA, especially using potent genome editing strategies, may be the basis for novel, highly effective options to adeptly and stably treat this incurable joint disorder.

## 4. Mitochondrial Genome Editing and Potential Treatments for Osteoarthritis

**Figure 2:**
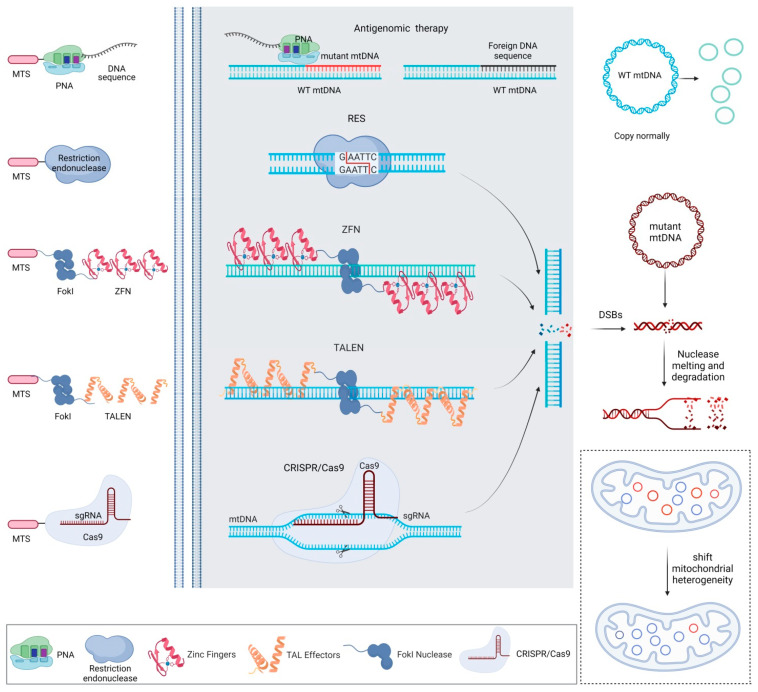
Mitochondrial genome editing strategies to target the mtDNA. The approaches include the use of antigenomic therapy, restriction endonucleases, ZFNs, TALENs and CRISPR/Cas9. Abbreviations: mtDNA, mitochondrial DNA; ZFNs, zinc-finger nucleases; TALENS, transcription activator-like effectors nucleases; CRISPR/Cas9, clustered regularly interspaced short palindromic repeats/CRISPR-associated 9; MTS, mitochondria-targeting sequence; PNA, peptide nucleic acid; sgRNA, single guide RNA; WT, wild-type; DSBs, double-strand breaks (created with BioRender).

Translationally adapted technologies and systems have been developed to edit the mitochondrial genome to manage mtDNA mutations and mitochondrial dysfunction, including antigenomic mtDNA therapy, restriction endonucleases, zinc-finger nucleases (ZFNs), transcription activator-like effectors nucleases (TALENs) and clustered regularly interspaced short palindromic repeats/CRISPR-associated 9 (CRISPR/Cas9) (Table 1 and Figure 2) [40,41,42,43,44,45,46,47,48,49,50,51,52,53,54,55,56,57,58,59,60,61,62,63,64,65,66,67,68,69,70,71,72,73,74,75,76,77,78,79,80,81,82,83,84].

The potential of antigenomic mtDNA therapy [40] was reported based on the use of peptide nucleic acids (PNAs) complementary to human mtDNA templates containing pathogenic deletion breakpoints and single-base mutations using mitochondria-targeting sequences (MTS) and triphenylphosphine to specifically inhibit the replication of the mutant sequences [41,42]. This therapy was also tested to reduce mtDNA *ND5* mutant genes in human cells using a 20-nucleotide sequence paired with a variant mtDNA region for mitochondrial point mutations [43].

Mitochondria-targeted restriction (bacterial) endonucleases have been employed to shift mtDNA heterogeneity and reduce the burden of mitochondrial genome mutations [44,45,46,47]. The PstI endonuclease was manipulated to shift mtDNA heterogeneity in rodent cells via accumulation of mtDNA phenotypes lacking PstI sites [48]. Xmal and SmaI were used to digest fragments in the mitochondrial T-to-G position 8993 (T8993G) mutation sequence affecting the *ATP 6* gene in models of Leigh syndrome (lacticacidemia, hypotonia, neurodegeneration), normalising the production of ATP as well as the mitochondrial membrane potential [30,49,50,51]. This strategy, however, is limited by the low number of mutations that can be targeted by these endonucleases and by the fact that it may affect the entire mtDNA, potentially causing its entire degradation [44].

ZFNs, composed of a specific DNA recognition domain with tandems of Cys_2_-His_2_ zinc-finger proteins and a sequence-independent endonuclease, cut specific sites in the form of dimers [52]. ZFNs were applied to target the T8993G mutation site in the mtDNA with an MTS by inserting the marker *hDNMT3a methylase* sequence as a tracer of specific mtDNA modification [53] and by shifting mtDNA heterogeneity to reduce or eliminate this mutation and to restore the mitochondrial respiratory functions in human and mammalian cells [54,55,56]. ZFNs recognising the C5024T mutation of mtDNA with an MTS were also employed to eliminate mutant mtDNA in the heart of a mouse model and to alleviate mitochondrial dysfunction in cardiac tissue [57]. Yet, while ZFNs can be used for almost all mtDNA predetermined sequences [54,56,58], their presence in the targets may raise cytotoxic responses, hindering their safe application in vivo [58,59].

TALENs, naturally secreted by plant pathogens of the Xanthomonas species, exhibit a DNA sequence recognition function by their amino acid combination with repeat variable two-residue (RVD) modules assigned with amino acids at positions 12 and 13 [60]. Four different RVD modules with customisable arrays of polymorphic amino acids (Asn-Asn, Asn-Ile, His-Asp and Asn-Gly) are the most widely used to identify guanine and adenine, cytosine and thymine, respectively [60]. When two TALENs monomers bind to DNA, initiating the formation of dimers, the functional FokI unit of the system breaks the double bond [61]. TALENS have been broadly employed since their DNA-binding specificity is easier to engineer than that of ZFNs [62,63]. TALENS fused to an MTS were employed to shift mitochondrial heterogeneity by eliminating the point mutations G14459A [64], 8483_13459del4977 [65], A8344G and G13513A [66,67,68] and A3243G [69], increasing complex VI activity and the mitochondrial oxidative phosphorylation capacity. Recent work also reported the efficacy of TALENs fused with split-DddA halves from the DddA interbacterial toxin and with a uracil glycosylase inhibitor to accurately manipulate mtDNA at the level of a single base, catalysing C•G to T•A conversions [70] instead of eliminating mtDNA copy numbers that may be potentially toxic in vivo. Such an approach may help better understand mtDNA mutations in human mtDNA-related diseases and conditions such as ageing and to treat or prevent primary mtDNA mutation diseases [71,72].

The discovery of the groundbreaking CRISPR/Cas9 technology by Emmanuelle Charpentier and Jennifer Doudna [73], recipients of the 2020 Nobel Prize in Chemistry, opens new avenues of research that may be adapted to treat mitochondrial diseases. CRISPR/Cas9, a bacterial immunoprotective system that affords resistance to foreign DNA, is composed of a sequence-dependent single guide RNA (sgRNA) and a Cas9 endonuclease. The sgRNA (a CRISPR RNA-crRNA—and trans-activating crRNA-tracrRNA) specifically recognises the base sequence of a target gene and instructs Cas9 to generate DNA double-strand breaks (DSBs) at protospacer adjacent motifs (PAMs) [74], allowing to develop therapeutic strategies for cancer (lung metastasis), blood disorders (haemoglobinopathies) or viral infections (human immunodeficiency virus—HIV) [75,76,77,78,79]. CRISPR/Cas9 has been employed to target the *Cox1* and *Cox3* loci in mtDNA, leading to mitochondrial membrane potential disruption and cell growth inhibition [80]. Yet, the use of this system for mtDNA editing remains challenging. First, the double-layer membrane of the mitochondria is a natural barrier to the sgRNA [81]. This issue might be addressed by using a system based on the Leishmania RNA import complex (RIC) capable of translocating tRNA in the mitochondria to normalise mitochondrial dysfunction caused by the mt-tRNA^Lys^ (MT-TK) mutation [82,83,84]. Next, the problems of the absence of DNA repair mechanisms in the mitochondria [85,86] and potential off-target effects need to be considered for mtDNA editing, although the heterogeneity and multi-copy characteristics of the mitochondria reserve fault-tolerant space for the biological therapy of mitochondrial gene editing.

As mitochondrial gene mutations are associated with the occurrence and progression of OA, manipulating or restoring the normal state of the mtDNA via genome editing procedures may provide potentially effective treatments to cure this disorder. While little is still known on the potential of mitochondrial genome editing in OA, strategies targeting mutated mitochondria genes contributing to OA-associated oxidation, or even genes involved in inflammation and/or ageing, may be envisaged by correcting the altered phenotypes or by transferring adapted anti-inflammatory, differentiation and/or regenerative gene sequences. Such edited mitochondria may further be used as immunopriviledged organelle transplants [87] for administration in OA targets as a means to enhance the efficacy of mtDNA editing in light of evidence showing that increasing the total amount of mtDNA improves the function of mitochondria and alleviates the symptoms of mitochondrial diseases [88]. Transplantation of edited mitochondria may be performed directly or in conjunction with clinically used, biocompatible materials to promote a sustained, controlled release of the organelles in a precise spatial and temporal manner in the OA joints [89,90].

## 5. Conclusions

Editing the mitochondrial genome is an emerging, attractive strategy to manage mutations in the mtDNA in OA and, subsequently, the dysfunction of the mitochondrial oxidative phosphorylation processes in OA chondrocytes that substantially contribute to the pathogenesis of this severe, highly prevalent human disorder. Mitochondrial genome editing applied to OA is still in its infancy, but such an innovative concept may be envisaged using all available editing tools, especially the CRISPR/Cas9 system. It may further be combined with the transplantation of edited (autologous or even allogeneic) mitochondria with inherent (and further improved) antioxidative abilities [91] to shift mitochondrial heterogeneity, to correct mtDNA mutations and to restore the oxidative functions to natural levels in OA cells (and patients) upon direct administration or used as cargos via controlled release (scaffold-guided) approaches. While a number of challenges remain to be addressed for the effective and safe application of genome editing procedures to target the mtDNA in OA, including the selection of an optimal editing system, the presence of barriers to effective editing in the mitochondria (double-layer membrane), the absence of mtDNA repair mechanisms and potential off-target effects, this strategy holds strong potential and value in translational applications to treat human OA in the future.

## Figures and Tables

**Table 1:** Genome editing technologies.

Types	Recognition Sites	Advantages	Limitations	Refs.
antigenomic mtDNA	antigenomic-BS	easy to design	large, limited target sites, poor specificity	[40,41,42,43]
REs	RE-BS	easy to design	large, limited target sites, poor specificity	[44,45,46,47,48,49,50,51]
ZFNs	ZFN-BS	small, highly specific	difficult to design, limited target sites	[52,53,54,55,56,57,58]
TALENs	TALEN-BS	easy to design, highly specific	large, limited target sites	[60,61,62,63,64,65,66,67,68,69,70,71,72]
CRISPR/Cas9	CRISPR/Cas9-BS	easy to design	gRNA import, limited target sites	[80,82,83,84]

Abbreviations: mtDNA, mitochondrial DNA; REs, restriction endonucleases; ZFNs, zinc-finger nucleases; TALENS, transcription activator-like effectors nucleases; CRISPR/Cas9, clustered regularly interspaced short palindromic repeats/CRISPR-associated 9; BS, binding sites.

## Data Availability

Not applicable.
